# Dietary Nutritional Level Affects Intestinal Microbiota and Health of Goats

**DOI:** 10.3390/microorganisms10122322

**Published:** 2022-11-24

**Authors:** Hongran Guo, Bibo Li, Meiqi Gao, Qian Li, Yawei Gao, Ning Dong, Gongwei Liu, Zhichao Wang, Wenrui Gao, Yulin Chen, Yuxin Yang

**Affiliations:** 1 Innovative Research Team of Sheep and Goat, College of Animal Science and Technology, Northwest A&F University, Xianyang 712100, China; 2 College of Animal Science, Shanxi Agricultural University, Jinzhong 030801, China; 3 Hengshan District Animal Husbandry Bureau, Yulin 719000, China

**Keywords:** dietary nutritional level, cecum, colon, microbiota, goats

## Abstract

The intestine is a complex micro-ecosystem, and its stability determines the health of animals. Different dietary nutritional levels affect the intestinal microbiota and health. In this study, the nutritional levels of energy and protein in the diet of goats were changed, and the body weight was measured every 15 days. In the late feeding period, 16 S rRNA sequencing technology was used to detect the content of microorganisms. A meteorological chromatograph was used to detect volatile fatty acids in the cecum and colon of goats. In the feeding stage, reducing the nutritional level of the diet significantly reduced the weight of the lamb (*p* < 0.05). In the cecum, the abundance of potentially harmful bacteria, such as *Sphingomonas*, *Marvinbryantia,* and *Eisenbergiella,* were significantly enriched in goats fed with the standard nutritional level diets (*p* < 0.05). Additionally, the contents of acetate (*p* = 0.037) and total VFAs (*p* = 0.041) increased. In the colon, the abundance of SCFAs-producing bacteria, such as *Ruminococcaceae*, *Christensenellaceae,* and *Papillibacter,* decreased as the nutritional level in the diet increased (*p* < 0.05). In conclusion, the increase in nutritional level could affect the growth performance and composition of intestinal microbiota.

## 1. Introduction

The hindgut of ruminants, such as cecum and colon, contains a large number of microorganisms. Microbiota has a close relationship with their host in a long-term evolutionary process [1]. Microbiota can ferment the substrates and produce secondary metabolites, affecting intestinal tissues [2]. The changes in dietary nutritional levels affect hosts by affecting the composition of gut microbiota [3,4].

Goats are fed with a high nutritional diet to obtain a high yield of animal products and improve feed efficiency during the fattening period. However, the intake of high-grain diets, insufficient degradation in the rumen, and excessive fermentation of carbohydrates cause the imbalance of gut microbiota, affecting the animals’ health and reducing their production performance. When goats are fed with high concentrations of grains, the hindgut microbiome is dysregulated, and the metabolism is disturbed. The harmful bacteria, such as *Turicibacter* and *Clostridium*, increased in both the cecal and colonic digests [5]. Long-term exposure of feed to a high proportion of concentrate causes the enrichment of *Streptophyta* and destroys the barrier in the jejunum [6].

However, in the hindgut, the digestion of nutrients is lower than that in the rumen, and the dietary energy provided by the hindgut fermentation reaches 5–10% [7]. The long-term feeding of ruminants with high-cereal diets leads to excessive fermentation in the hindgut, causing acidosis. Posterior intestinal acidosis is caused by impaired intestinal function. The posterior intestine is protected from pathogenic bacteria by the mucosal layer [8]. Therefore, the posterior intestine can be easily attacked by harmful bacteria, destroying epithelial integrity and function [9]. The pathogenic bacteria damage the structure of the hindgut epithelium, thereby affecting animals’ health [10]. However, the effects of changes in gut microbiota on metabolic status in goats at the different energy and protein levels of diet have not been studied under certain forage-to-concentrate ratios.

The cecum and colon absorb water in food residues. These are part of the immune system, which performs a role in defending against foreign bacteria and viruses, protecting the body from damage, and stabilizing cleaning and monitoring functions. In the gut, metabolites, such as short-chain fatty acids (SCFAs), organic amines, and endotoxins, are absorbed by the hosts and benefit their health or cause metabolic disorders [11]. The metabolite SCFAs promote intestinal epithelial cells to produce antimicrobial peptides via G protein-coupled receptor 43 (GPR43), preventing the destruction of intestinal tissues [12]. The addition of acetate to the diet inhibits the translocation of intestinal microbiota and infection of *Clostridium difficile* by activating innate immune response [13]. Lipopolysaccharides (LPS) may cause intestinal inflammation. An increase in the concentration of LPS in plasma destroys intestinal barrier function [14].

We adopted an approach that combines the microbiome and metabolome of hindgut digest and mucosa to investigate the effect on the metabolic status of goats under diets with increased energy and protein levels at certain forage-to-concentrate ratios. The different energy and protein levels could significantly alter the composition and structure of microbiota in the cecum and colon. The changes in gut microbiota might alter the metabolite contents. In this study, the effects of dietary nutrition level on the gastrointestinal health of animals were revealed by investigating the alterations in microbiota and metabolic status in goat cecum and colon. We aimed to provide a theoretical basis for rationally adding dietary nutrition levels and increasing the growth and health of goats.

## 2. Materials and Methods

### 2.1. Experimental Animals and Study Design

The study was conducted in the original breeding farm of northern Shaanxi white cashmere goats in Diqingyuan, Hengshan County, Yulin City, northern Shaanxi, China, in 2017. The feeding period was from July to November and lasted for 4 months. A total of 9 two-month-old healthy goats (Shaanbei white cashmere goat) with similar weights were selected. The goats were randomly divided into 3 groups (groups A, B, and C) (*n* = 3 in each group). The goats in groups A, B, and C were fed with experimental diets having digestive energy (DE) and crude protein (CP) levels of 85%, 100%, and 130% of the standard diet, respectively.

### 2.2. Experimental Diet and Feeding Management

The standard diet was formulated in accordance with the National Research Council (NRC) feeding standards and feeding standard of meat-producing sheep and goats of the Chinese agricultural industry standards (NY/T816-2004). Based on the laboratory’s previous results, DE was 9.06 MJ/kg, and CP level was 10.38% (Table S1). The experimental diets were prepared to have the DE and CP levels of 85%, 100%, and 130% of the standard diets, respectively, with a dietary concentrate-to-forage ratio of 4:6. The specific composition and nutritional level of diets are provided in Table S1. The test ratio was complete formula feed, and the same amount of feed was fed at 8:00 and 18:00 every day. There was a little excess feed and free drinking water. One week before starting the study, the experimental houses were disinfected, which were also regularly disinfected during the test experiments.

### 2.3. Sample Collection

During the test, the weight was weighed every 15 days, and the initial weight and the last weight were used to calculate the daily gain. On the last day of the experiments, the goats were sacrificed after 4 h of morning feeding. Then, their abdominal cavity was opened, and both the ends of the cecum and colon were tied tightly with sutures to avoid refluxing digestive materials to adjacent areas. The intestinal tract was cut lengthwise with sterile scissors, and the intestinal contents were collected with a sterile cryotube. The intestinal mucus was scraped with a sterile spatula and collected into sterile tubes. P^H^ was measured, and the samples from cecum and colon contents were homogenized and divided into 3 aliquots. The first aliquot was placed in a 5-mL cryotube (boopu, Chaoyang District, Beijing, China) and placed in liquid nitrogen for microbial genomic DNA (gDNA) extraction and analysis. To the second aliquot, water was added twice the volume of the aliquot and centrifuged at 2000× *g* for 10 min at 4 °C. Then, the supernatant was stored at −20 °C to determine the concentration of volatile fatty acids (VFAs). The last aliquot was mixed with an equal volume of sterile water and centrifuged immediately at 13,000× *g* for 40 min at 4 °C. The supernatant was filtered through a 0.22 μm filter (without endotoxin), placed in a sterile tube, and then stored at −20 °C to determine the endotoxin contents.

### 2.4. Microbial gDNA Extraction and Illumina-MiSeq Sequencing

The microbial gDNA extraction was carried out using the traditional bead mill and SDS/GITC/NH4Ac method [15]. Nucleic acid quantifier NanoDrop 1000 (Life Technologies, Carlsbad, CA, USA) was used to determine the concentration and purity of the extracted DNA, while its quality was assessed using 1.2% agarose (sial, Chaoyang District, Beijing, China) gel electrophoresis. The DNA samples that met the requirements were subjected to MiSeq sequencing.

The bacterial 16S rRNA gene universal primers (338F (5′-ACTCCTACGGGAGGCAGCAG-3′) and 806R (5′-GGACTACHVGGGTWTCTAAT-3′)) were used for sequencing. FLASH software was used to splice the original sequence, and FAETP was used for quality control. According to the 97% similarity level, UPARSE software was used to divide all the sequences into operational taxonomic units (OTUs). Based on OTU, α- and β-diversities of microbiota were analyzed. QIIME software was used to compare the OTU representative sequence with the data from RDP bacterial database. The composition of microbial communities of each sample at different classification levels (phyla, class, order, family, and genus) was identified, and the corresponding statistical analyses were performed. PICRUSt2 (phylogenetic investigation of communities by reconstruction of unobserved states 2) was used to predict the molecular function of microbiota in each sample.

### 2.5. Determination of Functional Bacteria Copy Number

Fluorescent quantitative real-time polymerase chain reaction (qRT-PCR) technology was used to detect the bacterial copy number by the absolute quantitative method. The functional strains were selected for quantitative detection and divided into three categories according to their functions: (1) *Ruminococcus albus 7*, *Ruminococcus flavefaciens FD-1*, *Butyrivibrio fibrisolvens,* and *Fibrobacter succinogenes S85*; (2) *Prevotella brevis GA33* and *Prevotella ruminicola 23*; and (3) *Ruminobacter amylophilus*, *Succinivibrio dextrinisolvens*, and *Selenomonas ruminantium D*. The quantitative method was carried out as described previously [15]. The primer sequences for these bacteria are provided in Table S2.

### 2.6. Determination of pH, VFAs, and Endotoxin (LPS) Content

The pH was measured using a portable pH meter (mettler toledo, Pilot Free Trade Zone, Shanghai, China). The contents of VFAs, including acetate, propionate, butyrate, valerate, iso-butyrate, and isovalerate, were measured using a high-performance meteorological chromatograph (Agilent Technologies 7820A GC system, Santa Clara, CA, USA) [16]. The Goat endotoxin enzyme-linked immunosorbent assay (ELISA) kit (Fankewei Company, Jinshan District, Shanghai, China) was used to determine the contents of LPS.

### 2.7. Statistical Analysis

Shapiro-Wilk and Bartlett tests in R software version 3.5.1 (R Core Team, Vienna, Austria) were used to test the normality and uniformity of the variance of residue distribution in all the data (*p* > 0.05). The test data was preliminarily processed using Excel 2016 and analyzed using the statistical software SPSS v24. As for the α diversity index, student’s *t*-test was used for analysis. Additionally, the analysis of variance was performed using one-way analysis of variance (ANOVA) and least significant difference (LSD) for two and multiple comparisons, respectively. The correlations of microbial species with intestinal metabolites were determined using Spearman’s correlation coefficient. *p* < 0.05 indicated a significant difference.

## 3. Results

### 3.1. Comparison of Growth Performance under Different Nutritional Levels

According to Figure S1, the average daily gain (ADG) of each group of sheep did not change significantly. On days 46 (*p* = 0.028), 61 (*p* = 0.026), and 76 (*p* = 0.041) of the test, the weight was significantly lower in group A compared with groups B and C, but the difference between groups B and C was not significant. At the final stage of the trial, there was no significant difference in weight among the three groups.

### 3.2. Comparison of Microbiota in the Cecum Digest under Different Nutritional Levels

The 16S rRNA gene sequencing was performed on the 9 samples of cecum contents. The dilution curve showed that all the samples were sequenced with sufficient depth and reached the plateau (Figure S2A). In the cecum digest samples, 18 phyla, 242 genera, and 1701 OTUs were found. The Shannon index decreased significantly in group C compared with group B (*p* = 0.003). The Simpson index increased significantly in group C compared with group B (*p* = 0.002) (Table S4). The principal coordinate analysis (PCoA) analysis based on the Bray–Curtis distance algorithm and analysis of similarities (ANOSIM) showed that group C was clearly distinguished from groups A and B in the cecum digest samples (Figure S2C).

**Figure 1:**
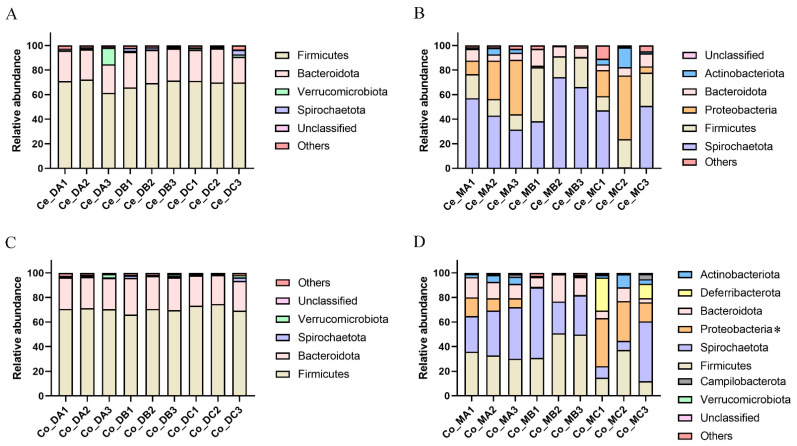
Effect of different nutritional levels on the abundance of bacteria in the phyla level (**A**) in the cecum digest, (**B**) cecum mucosa, (**C**) colon digest, (**D**) and colon mucosa. Bacteria with an abundance of less than 1% are classified as Others. “*” indicates 0.01 < *p* < 0.05.

**Figure 2:**
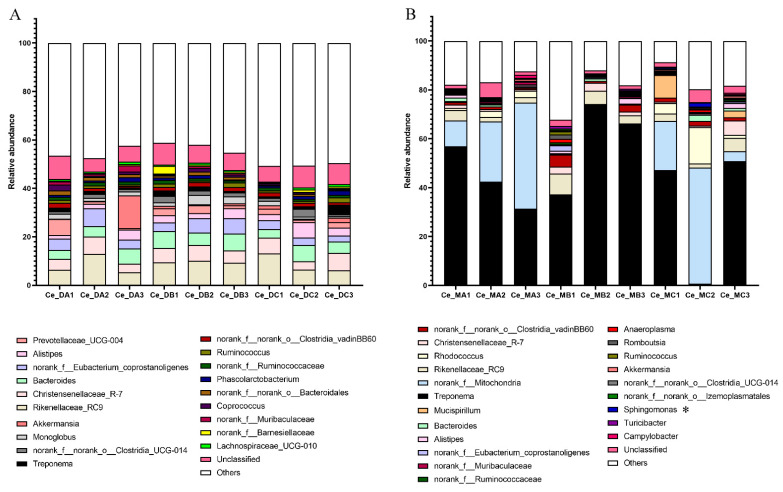
Effect of different nutritional levels on the abundance of bacteria in the genus level (**A**) in cecum digest, and (**B**) cecum mucosa. Bacteria with an abundance of less than 1% are classified as Others. “*” indicates 0.01 < *p* < 0.05.

Firmicutes was the most dominant phyla in the cecum digest (Figure 1A). At the genus level, in the cecum digest, the relative abundances of *Oscillibacter* were higher in group B compared with group A (*p* = 0.021). The abundance of *Marvinbryantia* was higher in group C compared with groups A and B (*p* = 0.046). The abundance of *Candidatus_Soleaferrea* was lower in group C compared with groups A and B (*p* = 0.032). The abundance of *Eisenbergiella* was lower in group A compared with groups B and C (*p* = 0.004) (Figure 2A and Table S5). Additionally, the diet had a significant effect on the copy number of *F. succinogenes S85* in the cecum digest samples, which decreased significantly in group C compared with group B (*p* = 0.016). The copy number of *P. brevis GA33* decreased significantly with the increase in energy and protein in the diet (*p* = 0.004) (Table S6).

### 3.3. Comparison of Microbiota in the Cecum Mucosa under Different Nutritional Levels

The 16S rRNA gene sequencing was performed on the nine samples of cecum mucosa. The dilution curve showed that all the samples were sequenced with sufficient depth and reached the plateau (Figure S2B). In the cecum mucosal samples, 32 phyla, 489 genera, and 2275 OTUs were found. The Sob (*p* = 0.048), Ace (*p* = 0.023), and Chao (*p* = 0.025) indexes decreased significantly in group C compared with group B (Table S7). The PCoA analysis based on the Bray–Curtis distance algorithm and ANOSIM showed that group C was clearly distinguished from groups A and B in the cecum mucosal samples (Figure S2D). *Spirochaetota* was the most dominant phyla in the cecum mucosa samples (Figure 1B). At the genus level, the relative abundance of *Sphingomonas* was higher in group C compared with group B (*p* = 0.029) (Figure 2B and Table S8).

### 3.4. Comparison of Microbiota in the Colon Digest under Different Nutritional Levels

The 16S rRNA gene sequencing was performed on the nine samples of colon contents. The dilution curve showed that all the samples were sequenced with sufficient depth and reached the plateau (Figure S3A). In the colon digest samples, 20 phyla, 240 genera, and 1684 OTUs were found. The Simpson index was significantly higher in group C compared with group A (*p* = 0.028) (Table S9). The PCoA analysis based on the Bray–Curtis distance algorithm showed that the samples in group C were distinguished from other groups in the colon digest (Figure S3C).

**Figure 3:**
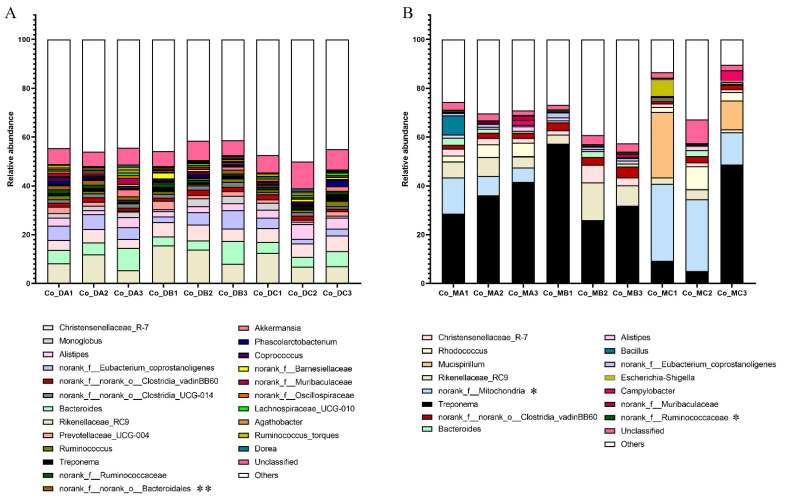
Effect of different nutritional levels on the abundance of bacteria in the genus level (**A**) in the colon digest and (**B**) colon mucosa. Bacteria with an abundance of less than 1% are classified as Others. “*” indicates 0.01< *p* <0.05. “**” indicates *p* < 0.01.

Firmicutes was the most dominant phylum in both the colon digest samples (Figure 1C). At the genus level, the abundance of *norank_f__norank_o__Bacteroidales* decreased significantly in group C compared with group A (*p* = 0.008). The abundance of *Candidatus_Saccharimonas* increased significantly in group A compared with groups B and C (*p* = 0.018) (Figure 3A and Table S10). Additionally, the diet had a significant effect on the copy number of cellulose-degrading bacteria in the colon samples. The copy number of *F. succinogenes* was significantly higher in group B (*p* = 0.015). The copy number of *R. albus 7* (*p* = 0.038) and *R. flavefaciens FD-1* (*p* = 0.011) was significantly higher in group C compared with group A (Table S11).

### 3.5. Comparison of Microbiota in the Colon Mucosa under Different Nutritional Levels

The 16S rRNA gene sequencing was performed on the nine samples of the colon mucosa. The dilution curve showed that all the samples were sequenced with sufficient depth and reached the plateau (Figure S3B). In the colon mucosal samples, 31 phyla, 501 genera, and 2362 OTUs were found. The Ace index was higher in group B compared with group A (*p* = 0.037) and C (*p* = 0.024). The Chao index was higher in group B compared with groups A (*p* = 0.028) and C (*p* = 0.023) (Table S12). The PCoA analysis based on the Bray–Curtis distance algorithm showed that the samples were clearly distinguished from each other among the groups (Figure S3D).

Firmicutes was the most dominant phylum in both the colon mucosa samples. The abundance of Proteobacteria (*p* = 0.037) was higher in group C (Figure 1D). At the genus level, the abundance of *norank_f__Mitochondria* was lower in group B compared with group C (*p* = 0.045). The relative abundance of *norank_f__Ruminococcaceae* was higher in group B compared with group C (*p* = 0.020). The relative abundance of *norank_f__norank_o__Izemoplasmatales* decreased significantly in group C compared with groups A and B (*p* = 0.019). The relative abundance of *unclassified_f__Ruminococcaceae* (*p* = 0.032), *Papillibacter* (*p* = 0.033), and *norank_f__Christensenellaceae* (*p* = 0.004) were higher significantly in group B. The relative abundance of *Coprococcus* decreased significantly in group C compared with group A (*p* = 0.034) (Figure 3B and Table S13).

### 3.6. Effects of Dietary Nutritional Level on Intestinal pH, VFAs, and LPS

**Figure 4:**
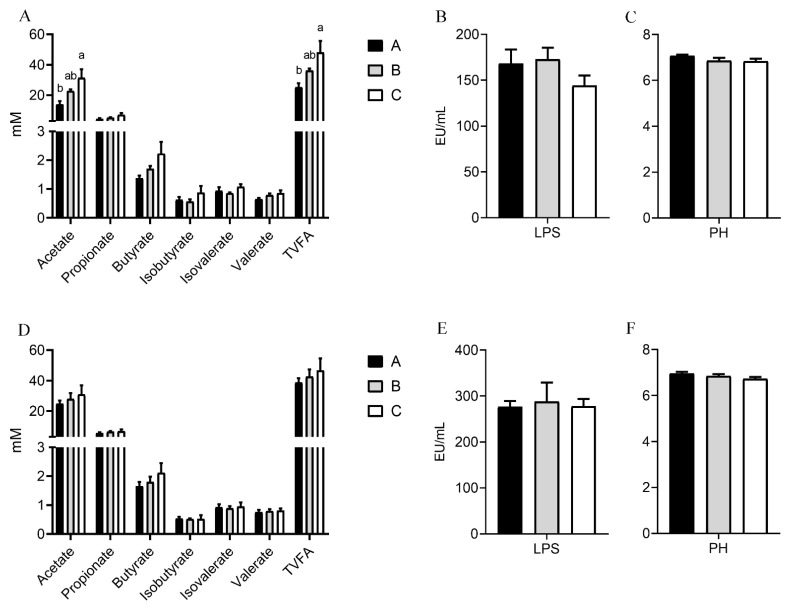
Effect of different nutrition levels on pH, VFAS, and LPS in the cecum (**A**–**C**) and colon (**D**–**F**). (**A**) Concentration of VFAs in cecum (**B**) Concentration of LPS in cecum (**C**) PH in cecum (**D**) Concentration of VFAs in colon (**E**) Concentration of LPS in colon (**F**) PH in colon. Different letters ^a, b^ represented significant differences between groups.

In the cecum, the contents of acetate (*p* = 0.037) and total VFAs (*p* = 0.041) increased as the energy and protein levels of the diet increased (Figure 4). However, the dietary nutritional level had no significant effects on the pH, VFAs, and LPS in the colon.

## 4. Discussion

This study was conducted to investigate the effects of different dietary energy and protein levels on growth performance and microbiota in the cecum and colon. The composition of gut microbiota altered when the goats were fed with different nutritional level diets. The abundance of potentially harmful bacteria, such as *Sphingomonas*, increased when the goats were fed with a high-nutritional diet. Meanwhile, the abundance of SCFAs-producing bacteria, such as *Ruminococcaceae*, *Christensenellaceae,* and *Papillibacter*, decreased. The intestinal microbiota was affected when the goats were fed with a high-nutritional diet.

The results of the previous article showed that reducing the energy and protein levels in the diet would reduce growth performance [17], which is consistent with our experimental results. However, the final weight was not significantly different after 120 days of feeding. The low CP diet has the same or higher feed consumption trend compared with the normal protein diet. The dietary energy and protein levels had little effect on body weight [18].

The composition of gut microbiota altered in the cecum with the increase in energy and protein levels in diets. The α diversity index (Sobs, Ace, and Chao) increased significantly in the cecum when 7% defatted rice bran (DFRB) fiber was added to the feed of Suhuai Pigs [19], which is consistent with our results. Diets had a lower fiber content and a lower α diversity index in group C. The abundance of bacteria related to inflammation also increased when the energy and protein levels of diets improved. *Marvinbryantia* can produce butyrate and increase the possibility of inflammation in the gut [20]. *Eisenbergiella* was enriched in the intestine of people fed with a low-fiber diet and might be related to the development of chronic diseases [21]. *Sphingomonas*, a pathogenic bacterium, was also enriched in the colonic mucosa [22]. These changes implied that long-term feeding of high nutrient levels might inflame the cecum and damage the cecal barrier function. When animals were fed with a small proportion of fiber, the abundance of inflammation-related bacteria also increased [23]. Additionally, the abundance of bacteria that degrade nutrients also decreased significantly when the energy and protein levels of diets were improved. *F. succinogenes* is a strictly anaerobic cellulolytic bacterial species, which uses cellulose and glucose [24]. The decrease in the abundance of *Candidatus_Soleaferrea* is correlated with nitrogen utilization [25]. *Prevotella* decomposes protein and carbohydrates [26] and decreases in abundance as dietary protein and energy levels decrease.

The composition of the microbiota is altered in the colon with alterations in energy and protein levels in the diet. The abundance of SCFAs-producing bacteria decreased when the goats were fed with a high-energy diet. Similarly, a low-fiber diet reduces short-chain fatty acids [27]. The abundance of *norank_f__norank_o__Bacteroidales* is related to acetate production [28]. *Norank_f__Ruminococcaceae* can degrade carbohydrates or oligosaccharides to produce SCFAs [29]. *Papillibacter* is a butyrate-producing bacterium [30]. Meanwhile, the abundance of *Candidatus saccharimonas* is related to the biosynthesis of amino acids [31]. *Christensenellaceae* increases the diversity of intestinal microbiota and reduces fat deposition [32]. The abundance of these potential probiotics decreased. *R. flavefaciens FD-1* is a major fiber-degrading bacterium present in the intestines of herbivores and has a very complex cellulosic tissue [33]. However, *R. flavefaciens FD-1* can easily occupy the niche when the cellulose content is low.

Nutrition level influences the intestinal microbiota. When the crude protein level in the diet increase, the protein cannot be completely digested and absorbed in the small intestine and then will reach the large intestine. The hindgut bacteria will be corrupted, and some harmful bacteria, such as *Marvinbryantia*, *Eisenbergiella,* and *Sphingomonas*, can be produced, affecting the intestinal tract. Higher levels of energy and protein in the diet indicate a lower percentage of fiber. Undigested cellulose and hemicellulose in the rumen are fermented and metabolized by bacteria after reaching the colon to produce volatile fatty acids. When the proportion of cellulose in the diet decreased, the cellulose entering the hindgut decreased, and the abundance of SCFAs-producing bacteria decreased. The change in intestinal microbiota may also affect growth performance.

In the cecum, the total VFAs increased when the rabbits’ feeding was changed from a fed low-protein diet to a high-protein diet [34]. Piglets fed high protein levels also had higher abundances of acetate in the cecum [35]. Similar results were obtained in this study. The VFAs are absorbed by the host for normal life activities. In addition to nutrition, the VFAs also regulate the physiological activities of animals. Acetate can promote the differentiation of B1a cells into B10 cells, thereby performing an anti-inflammatory role in humans and mice [36]. Increased acetic acid may provide relief from potentially harmful bacteria in the gut. LPS, a structural component of the cell wall of Gram-negative bacteria, is released after apoptosis and degradation. LPS in the intestines may cause inflammation or play an immunomodulatory effect [37]. The mice fed with a high-protein diet could exacerbate the severity of inflammatory bowel disease [38], and those fed with a high-fat diet could promote neuroinflammation [39]. In this study, the content of LPS did not change significantly and did not damage the intestinal tissues when the goats were fed with high-nutrient diets.

The change of bacteria may affect intestinal barrier integrity. In the cecum, *Sphingomonas* may cause intestinal inflammation by destroying the tight junctions. However, the increase in the content of acetate may alleviate this damage. In the colon, SCFAs-producing bacteria, such as *Ruminococcaceae*, *Christensenellaceae,* and *Papillibacter*, may have a beneficial influence on the tight junctions by SCFAs, and increase the expression levels of zonula occludens-1 (ZO-1), claudin, and occludin [40]. The increased abundance of harmful bacteria and decrease in SCFAs-producing bacteria might destroy the intestinal barrier’s integrity, leading to gut inflammation. Due to the small number of genes detected using 16S rRNA gene sequencing and correlation analysis, little information was found. *Sphingomonas* and SCFAs-producing bacteria should be cultured to investigate their contribution to intestinal health in further studies. In addition, we should use animal experiments to explore the effect of food intake on intestinal microbes in lambs.

## 5. Conclusions

In this study, the reduction in nutrition level during feeding affected growth performance. With the increase in dietary nutrition, in the cecum, the inflammation-related bacteria increased, but the content of acetate and total VFAs increased. In addition, the SCFAs-producing bacteria decreased in the colon. This study revealed that the growth performance and intestinal microbiota of goats changed significantly with different nutrition levels.

## Data Availability

Raw Illumina sequencing data have been deposited in Sequence Read Archive (SRA). The BioProject’s metadata is available at the following link: http://www.ncbi.nlm.nih.gov/bioproject/760658 (accessed on 1 January 2020).

## Supplementary Materials

The following supporting information can be downloaded at: https://www.mdpi.com/article/10.3390/microorganisms10122322/s1. Table S1: Composition and nutrient levels of the diet (air-dry basis). Table S2: The primer list of functional bacterial species in real-time PCR. Table S3: The primer list for real-time PCR. Table S4: Compare α diversity of the cecal digest under different nutrition levels. Table S5: Changes in the abundance of microbiota in the cecum digest (%). Table S6: Comparison of the copy number of functional bacterial species in cecum under different nutrition levels. Table S7: Compare α diversity of the caecum mucosa under different nutrition levels. Table S8: Changes in the abundance of microbiota in the cecum mucosa (%). Table S9: Compare α diversity of the colon digests under different nutrition levels. Table S10: Changes in the abundance of microbiota in the colon digest (%). Table S11: Comparison of the copy number of functional bacterial species in colon under different nutrition levels. Table S12: Compare α diversity of the colon mucosa under different nutrition levels. Table S13. Changes in the abundance of microbiota in the colon mucosa (%). Figure S1: Effects of different feeding rates on growth performance. (**A**) Average daily gain (ADG), (**B**) Lamb weight at different times. Figure S2: Refraction curves of bacteria in the (**A**) cecum digest and (**B**) cecum mucosa. PCoA of the microbial community in the (**C**) cecum digest and (**D**) cecum mucosa. Figure S3: Refraction curves of bacteria in the (**A**) colon digest and (**B**) colon mucosa. PCoA of the microbial community in the (**C**) colon digest and (**D**) colon mucosa.

## References

[1] Macfarlane G.T., Macfarlane S. (2012). Bacteria, colonic fermentation, and gastrointestinal health. J. AOAC Int..

[2] Chen F., Stappenbeck T.S. (2019). Microbiome control of innate reactivity. Curr. Opin. Immunol..

[3] Qu D., Wang G., Yu L., Tian F., Chen W., Zhai Q. (2021). The effects of diet and gut microbiota on the regulation of intestinal mucin glycosylation. Carbohydr. Polym..

[4] Shan K., Qu H., Zhou K., Wang L., Zhu C., Chen H., Gu Z., Cui J., Fu G., Li J. (2019). Distinct Gut Microbiota Induced by Different Fat-to-Sugar-Ratio High-Energy Diets Share Similar Pro-obesity Genetic and Metabolite Profiles in Prediabetic Mice. mSystems.

[5] Tao S., Tian P., Luo Y., Tian J., Hua C., Geng Y., Cong R., Ni Y., Zhao R. (2017). Microbiome-Metabolome Responses to a High-Grain Diet Associated with the Hind-Gut Health of Goats. Front. Microbiol..

[6] Jia J., Liang C., Wu X., Xiong L., Bao P., Chen Q., Yan P. (2021). Effect of high proportion concentrate dietary on Ashdan Yak jejunal barrier and microbial function in cold season. Res. Veter. Sci..

[7] Steele M.A., AlZahal O., Hook S.E., Croom J., McBride B.W. (2009). Ruminal acidosis and the rapid onset of ruminal parakeratosis in a mature dairy cow: A case report. Acta Veter. Scand..

[8] Caruso R., Lo B.C., Núñez G. (2020). Host–microbiota interactions in inflammatory bowel disease. Nat. Rev. Immunol..

[9] Tao S., Duanmu Y., Dong H., Ni Y., Chen J., Shen X., Zhao R. (2014). High Concentrate Diet Induced Mucosal Injuries by Enhancing Epithelial Apoptosis and Inflammatory Response in the Hindgut of Goats. PLoS ONE.

[10] Tovaglieri A., Sontheimer-Phelps A., Geirnaert A., Prantil-Baun R., Camacho D.M., Chou D.B., Jalili-Firoozinezhad S., de Wouters T., Kasendra M., Super M. (2019). Species-specific enhancement of enterohemorrhagic *E. coli* pathogenesis mediated by microbiome metabolites. Microbiome.

[11] Roewe J., Stavrides G., Strueve M., Sharma A., Marini F., Mann A., Smith S.A., Kaya Z., Strobl B., Mueller M. (2020). Bacterial polyphosphates interfere with the innate host defense to infection. Nat. Commun..

[12] Zhao Y., Chen F., Wu W., Sun M., Bilotta A.J., Yao S., Xiao Y., Huang X., Eaves-Pyles T.D., Golovko G. (2018). GPR43 mediates microbiota metabolite SCFA regulation of antimicrobial peptide expression in intestinal epithelial cells via activation of mTOR and STAT3. Mucosal Immunol..

[13] Fachi J.L., Sécca C., Rodrigues P.B., De Mato F.C.P., Di Luccia B., Felipe J.D.S., Pral L.P., Rungue M., Rocha V.D.M., Sato F.T. (2019). Acetate coordinates neutrophil and ILC3 responses against C. difficile through FFAR2. J. Exp. Med..

[14] Tulkens J., Vergauwen G., Van Deun J., Geeurickx E., Dhondt B., Lippens L., De Scheerder M.-A., Miinalainen I., Rappu P., De Geest B.G. (2020). Increased levels of systemic LPS-positive bacterial extracellular vesicles in patients with intestinal barrier dysfunction. Gut.

[15] Li B., Zhang K., Li C., Wang X., Chen Y., Yang Y. (2019). Characterization and Comparison of Microbiota in the Gastrointestinal Tracts of the Goat (*Capra hircus*) during Preweaning Development. Front. Microbiol..

[16] Guo H., Zhou G., Tian G., Liu Y., Dong N., Li L., Zhang S., Chai H., Chen Y., Yang Y. (2021). Changes in Rumen Microbiota Affect Metabolites, Immune Responses and Antioxidant Enzyme Activities of Sheep under Cold Stimulation. Animals.

[17] Li X., Li Q., Cao Y., Gao Y., Yu C., Du L., Wang X., Li J. (2015). Effects of dietary energy and protein levels on growing-fattening performance and blood biochemical indicators of holstein bulls. Chin. J. Anim. Nutr..

[18] Le Bellego L., van Milgen J., Noblet J. (2002). Effect of high temperature and low-protein diets on the performance of growing-finishing pigs. J. Anim. Sci..

[19] Pu G., Li P., Du T., Niu Q., Fan L., Wang H., Liu H., Li K., Niu P., Wu C. (2020). Adding Appropriate Fiber in Diet Increases Diversity and Metabolic Capacity of Distal Gut Microbiota without Altering Fiber Digestibility and Growth Rate of Finishing Pig. Front. Microbiol..

[20] Jiang Q., He X., Zou Y., Ding Y., Li H., Chen H. (2018). Altered gut microbiome promotes proteinuria in mice induced by Adriamycin. AMB Express.

[21] Bailén M., Bressa C., Martínez-López S., González-Soltero R., Lominchar M.G.M., Juan C.S., Larrosa M. (2020). Microbiota Features Associated with a High-Fat/Low-Fiber Diet in Healthy Adults. Front. Nutr..

[22] White D.C., Sutton S.D., Ringelberg D.B. (1996). The genus Sphingomonas: Physiology and ecology. Curr. Opin. Biotechnol..

[23] Sun Y., Zhang S., Nie Q., He H., Tan H., Geng F., Ji H., Hu J., Nie S. (2022). Gut firmicutes: Relationship with dietary fiber and role in host homeostasis. Crit. Rev. Food Sci. Nutr..

[24] Matheron C., Delort A.-M., Gaudet G., Forano E. (1997). Re-investigation of glucose metabolism in Fibrobacter succinogenes, using NMR spectroscopy and enzymatic assays.: Evidence for pentose phosphates phosphoketolase and pyruvate formate lyase activities. Biochim. et Biophys. Acta.

[25] Wang L., Liu K., Wang Z., Bai X., Peng Q., Jin L. (2019). Bacterial Community Diversity Associated with Different Utilization Efficiencies of Nitrogen in the Gastrointestinal Tract of Goats. Front. Microbiol..

[26] Christensen L., Vuholm S., Roager H.M., Nielsen D.S., Krych L., Kristensen M., Astrup A., Hjorth M.F. (2019). Prevotella Abundance Predicts Weight Loss Success in Healthy, Overweight Adults Consuming a Whole-Grain Diet Ad Libitum: A Post Hoc Analysis of a 6-Wk Randomized Controlled Trial. J. Nutr..

[27] Fettig N.M., Robinson H.G., Allanach J.R., Davis K.M., Simister R.L., Wang E.J., Sharon A.J., Ye J., Popple S.J., Seo J.H. (2022). Inhibition of Th1 activation and differentiation by dietary guar gum ameliorates experimental autoimmune encephalomyelitis. Cell Rep..

[28] Zhang Y., Xu Y., Chen X., Chen C., Sun J., Bai X., Yuan Y. (2021). Effect of copper ions on glucose fermentation pathways in bioelectrochemical system. Chemosphere.

[29] Wu Y., Zhang X., Tao S., Pi Y., Han D., Ye H., Feng C., Zhao J., Chen L., Wang J. (2020). Maternal supplementation with combined galactooligosaccharides and casein glycomacropeptides modulated microbial colonization and intestinal development of neonatal piglets. J. Funct. Foods.

[30] Bordoni L., Gabbianelli R., Fedeli D., Fiorini D., Bergheim I., Jin C.J., Marinelli L., Di Stefano A., Nasuti C. (2019). Positive effect of an electrolyzed reduced water on gut permeability, fecal microbiota and liver in an animal model of Parkinson’s disease. PLoS ONE.

[31] Ogunade I., Schweickart H., McCoun M., Cannon K., McManus C. (2019). Integrating 16S rRNA Sequencing and LC–MS-Based Metabolomics to Evaluate the Effects of Live Yeast on Rumen Function in Beef Cattle. Animals.

[32] Li X., Li Z., He Y., Li P., Zhou H., Zeng N. (2020). Regional distribution of *Christensenellaceae* and its associations with metabolic syndrome based on a population-level analysis. PeerJ.

[33] Rincon M.T., Dassa B., Flint H.J., Travis A.J., Jindou S., Borovok I., Lamed R., Bayer E.A., Henrissat B., Coutinho P.M. (2010). Abundance and Diversity of Dockerin-Containing Proteins in the Fiber-Degrading Rumen Bacterium, Ruminococcus flavefaciens FD-1. PLoS ONE.

[34] Trocino A., Fragkiadakis M., Majolini D., Tazzoli M., Radaelli G., Xiccato G. (2013). Soluble fibre, starch and protein level in diets for growing rabbits: Effects on digestive efficiency and productive traits. Anim. Feed Sci. Technol..

[35] Manzanilla E.G., Pérez J.F., Martin M., Blandón J.C., Baucells F., Kamel C., Gasa J. (2009). Dietary protein modifies effect of plant extracts in the intestinal ecosystem of the pig at weaning1. J. Anim. Sci..

[36] Daïen C., Tan J., Audo R., Mielle J., Quek L., Krycer J., Angelatos A., Duraes M., Pinget G., Ni D. (2021). Gut-derived acetate promotes B10 cells with antiinflammatory effects. JCI Insight.

[37] Di Lorenzo F., De Castro C., Silipo A., Molinaro A. (2019). Lipopolysaccharide structures of Gram-negative populations in the gut microbiota and effects on host interactions. FEMS Microbiol. Rev..

[38] Ng S.C., Ananthakrishnan A.N. (2018). New approaches along the IBD course: Diet, tight control and stem cells. Nat. Rev. Gastroenterol. Hepatol..

[39] Kim J.D., Yoon N.A., Jin S., Diano S. (2019). Microglial UCP2 Mediates Inflammation and Obesity Induced by High-Fat Feeding. Cell Metab..

[40] Tian P., Zhu H., Qian X., Chen Y., Wang Z., Zhao J., Zhang H., Wang G., Chen W. (2021). Consumption of Butylated Starch Alleviates the Chronic Restraint Stress-Induced Neurobehavioral and Gut Barrier Deficits through Reshaping the Gut Microbiota. Front. Immunol..

