# Supplementary material for: Dietary Nutritional Level Affects Intestinal Microbiota and Health of Goats
Source: Microorganisms. 2022 Nov 24;10(12):2322. doi: 10.3390/microorganisms10122322 (PMC9781347; doi:10.3390/microorganisms10122322)
Supplement: Supplementary file 1 [file microorganisms-10-02322-s001.zip › microorganisms-2050080-supplementary.pdf]

microorganisms

Article

Dietary nutritional level changes intestinal microbiota and affects epithelial barrier function

Hongran Guo [1†], Bibo Li [2†], Meiqi Gao [1], Kaixin Yuan [1], Ning Dong [1], Gongwei Liu [1], Zhichao Wang [1], Wenrui Gao [3], Yulin Chen [1], Yuxin Yang [1*]

1Innovative Research Team of Sheep and Goat, College of Animal Science and Technology, Northwest A&F University, Yangling 712100, Shaanxi Province, China
2College of Animal Science, Shanxi Agricultural University, Jinzhong 030801, Shanxi Province, China
3Hengshan District Animal Husbandry Bureau, Yulin, 719000, Shaanxi Province, China.
†Hongran Guo and Bibo Li contributed equally to this study.
* Correspondence: Yuxin Yang. Email: yangyuxin2002@126.com

Supplementary Table

Table S1. Composition and nutrient levels of the diet (air-dry basis).

Items	A	B	C
Ingredients			
Alfalfa meal	0.00	10.00	49.00
Corn straw	61.50	50.00	9.20
Corn	4.00	23.60	32.00
Wheat bran	28.00	6.00	0.00
Soybean oil	0.00	0.00	3.00
Soybean meal	0.00	2.96	5.00
Cottonseed meal	2.00	0.56	0.00
Rapeseed meal	2.25	5.00	0.00
CaCO3	0.75	0.09	0.00
CaHPO4	0.00	0.30	0.30
NaCl	0.50	0.50	0.50
Premix	1.00	1.00	1.00
Total	100.00	100.00	100.00
Nutrient level			
DM/%	88.80	91.30	89.70
DE/(MJ/kg)	8.06	9.03	11.51
CP/%	9.10	10.38	13.47
Ca/%	0.55	0.47	0.70
TP/%	0.38	0.35	0.38
EE/%	2.31	2.37	3.84
NDF/%	43.33	42.38	38.25
ADF/%	27.69	23.13	18.95

Note: The premix provided the following staff for per kg diet: VA 1 000 IU, VD 200 IU, VE 20 IU, Fe 40 mg, Zn 30 mg, Cu 15 mg, I 2 mg, Mn 40 mg.

All nutrient content data were measured values except DE were calculated value.

microorganisms

Table S2. The primer list of functional bacterial species in real-time PCR.

Category	Primer sequence (5′-3′)	Product size (bp)
Butyrivibrio fibrisolvens	ACCGCATAAGCGCACGGA<br>CGGGTCCATCTTGTACCGATAAAT	64
Fibrobacter succinogenes S85	GTTCGGAATTACTGGGCGTAAA<br>CGCCTGCCCCTGAACTATC	121
Ruminococcus albus 7	TGTTAACAGAGGGAAGCAAAGCA<br>TGCAGCCTACAATCCGAACTAA	75
Ruminococcus flavefaciens FD-1	TGGCGGACGGGTGAGTAA<br>TTACCATCCGTTTCCAGAAGCT	71
Prevotella brevis GA33	AGCGCAGGCCGTTTGG<br>GCTTCCTGTGCACTCAAGTCTGAC	91
Prevotella ruminicola 23	GAAAGTCGGATTAATGCTCTATGTTG<br>CATCCTATAGCGGTAAACCTTTGG	74
Ruminobacter amylophilus	CTGGGGAGCTGCCTGAATG<br>GCATCTGAATGCGACTGGTTG	102
Succinivibrio dextrinosolvens	TAGGAGCTTGTGCGATAGTATGG<br>CTCACTATGTCAAGGTCAGGTAAGG	174
Selenomonas ruminantium D	CAATAAGCATTCCGCCTGGG<br>TTCACTCAATGTCAAGCCCTGG	133

Table S3. The primer list for real-time PCR.

Primers	Sequences	Products size/ bp
β-actain F	ATATTGCTGCGCTCGTGGTT	211
β-actain R	CCGTGCTCAATGGGGTACTT	
Claudin-1 F	GATGCGAATGGCTGTCTTTG	159
Claudin-1 R	CCAATGAAGAGAGCCTGACC	
Claudin-4 F	GTATCATCCTGGCCGTGTTT	181
Claudin-4 R	AAGTCACGGATGACGTTGTT	
Occludin F	GGAGGCAGCATTAACTACCC	204
Occludin R	TATGCCTGACCTTACAACGC	
ZO-1 F	AAGTGGAACGTGGCATCATT	218
ZO-1 R	TTCTCGAAGAACCACCCTCT	

Table S4. Compare α diversity of the cecal digest under different nutrition levels.

Team	Ce_DA	Ce_DB	Ce_DC	*P* value(B-C)	*P* value(A-B)	*P* value(A-C)
Sobs	1002±97.22	1009±38.42	908.0±73.55	0.102	0.917	0.250
Shannon	5.217±0.293	5.409±0.086	5.068±0.019	0.003	0.338	0.429
Simpson	0.018±0.007	0.013±0.002	0.024±0.002	0.002	0.285	0.237
Ace	1152±77.07	1152±51.56	1042±75.90	0.107	0.997	0.153
Chao	1159±80.66	1198±49.43	1053±86.43	0.066	0.521	0.195
Coverage	0.994±0.000	0.994±0.000	0.995±0.001	0.264	0.857	0.184

Table S5. Changes in the abundance of microbiota in the cecum digest (%).

Heading	Ce_DA	Ce_DB	Ce_DC	P-value
Phylum				
Firmicutes	68.30±5.898	68.87±2.790	70.24±0.703	0.705
Bacteroidota	24.14±0.906	27.28±1.445	24.72±3.470	0.116
Verrucomicrobiota	4.679±7.665	0.769±0.355	1.215±0.718	0.563
Spirochaetota	1.119±0.445	1.553±0.789	1.877±1.886	0.681
Genus				
Oscillibacter	0.276±0.057[b]	0.495±0.033[a]	0.420±0.082[ab]	0.021
Marvinbryantia	0.364±0.017[b]	0.279±0.040[b]	0.532±0.090[a]	0.046
Candidatus_Soleaferrea	0.398±0.052[a]	0.426±0.033[a]	0.252±0.054[b]	0.032
Eisenbergiella	0.015±0.013[a]	0.068±0.016[b]	0.101±0.010[b]	0.004

Abbreviations: Ce_DA = Bacteria in the cecum digestion of goats in group A; Ce_DB = Bacteria in the cecum digestion of goats in group B; Ce_DC = Bacteria in the cecum digestion of goats in group C.
At the phylum level, at least one group of bacteria listed has an abundance greater than 1%.
At the genus level, the bacteria has a significant change and at least one group has an abundance greater than 0.1%.
Values are expressed as Mean ± Standard Deviation.
"[a, b, c]" represent significant changes. Different letters represent significant changes between groups.

Table S6. Comparison of the copy number of functional bacterial species in cecum under different nutrition levels

(lg(copies/g)).

Team	Ce_DA	Ce_DB	Ce_DC	*P* value
Butyrivibrio fibrisolvens	6.792±0.279	6.835±0.137	7.316±0.202	0.068
Fibrobacter succinogenes S85	6.075±0.566	7.158±0.576	4.743±0.918	0.016
Ruminococcus albus 7	7.089±0.198	7.287±0.449	7.147±0.129	0.709
Ruminococcus flavefaciens FD-1	7.578±0.697	7.852±0.233	7.898±0.173	0.644
Prevotella brevis GA33	6.101±0.036	5.825±0.210	5.505±0.079	0.004
Prevotella ruminicola 23	6.039±0.162	6.110±0.365	6.653±0.651	0.253
Ruminobacter amylophilus	5.057±0.345	5.117±0.162	5.060±0.528	0.976
Succinivibrio dextrinosolvens	6.838±0.124	6.934±0.199	7.479±0.898	0.352
Selenomonas ruminantium D	7.450±0.306	7.810±0.483	7.460±0.200	0.412

Table S7. Compare α diversity of the caecum mucosa under different nutrition levels.

Team	Ce_MA	Ce_MB	Ce_MC	*P* value(B-C)	*P* value(A-B)	*P* value(A-C)
Sobs	674.7±146.6	821.3±129.6	537.7±116.7	0.048	0.264	0.274
Shannon	2.911±0.303	3.263±0.970	3.083±0.416	0.783	0.581	0.593
Simpson	0.194±0.046	0.212±0.111	0.184±0.056	0.722	0.813	0.825
Ace	765.4±179.2	1047±135.6	599.8±166.6	0.023	0.095	0.306
Chao	776.3±181.0	1038±157.3	601.5±149.6	0.025	0.131	0.267
Coverage	0.995±0.002	0.992±0.000	0.997±0.002	0.012	0.027	0.330

Table S8. Changes in the abundance of microbiota in the cecum mucosa (%).

Team	Ce_MA	Ce_MB	Ce_MC	P-value
Phylum				
Spirochaetota	43.91±12.72	59.63±18.80	32.96±27.78	0.458
Firmicutes	15.25±3.997	28.23±13.78	20.39±8.060	0.385
Proteobacteria	28.86±16.72	0.522±0.717	26.10±23.57	0.129
Bacteroidota	6.668±2.355	9.971±3.245	7.425±2.984	0.482
Actinobacteriota	3.170±2.102	0.143±0.112	7.261±7.753	0.180
Deferribacterota	0.149±0.243	0.013±0.016	4.052±4.832	0.421
Genus				
Sphingomonas	0.159±0.043[ab]	0.002±0.004[b]	0.698±0.761[a]	0.029

Abbreviations: Ce_MA = Bacteria in the cecum digestion of goats in group A; Ce_MB = Bacteria in the cecum digestion of goats in group B; Ce_MC = Bacteria in the cecum digestion of goats in group C.
At the phylum level, at least one group of bacteria listed has an abundance greater than 1%.
At the genus level, the bacteria has a significant change and at least one group has an abundance greater than 0.1%.
Values are expressed as Mean ± Standard Deviation.
"[a, b, c]" represent significant changes. Different letters represent significant changes between groups.

Table S9. Compare α diversity of the colon digests under different nutrition levels.

Team	Co_DA	Co_DB	Co_DC	*P* value(B-C)	*P* value(A-B)	*P* value(A-C)
Sobs	986.3±113.7	995.3±77.98	886.7±88.75	0.186	0.915	0.298
Shannon	5.260±0.166	5.289±0.328	5.041±0.076	0.271	0.896	0.107
Simpson	0.017±0.003	0.017±0.008	0.024±0.002	0.204	0.990	0.028
Ace	1141±139.9	1142±71.55	1026±102.8	0.184	0.997	0.314
Chao	1115±140.9	1179±46.45	1042±98.88	0.096	0.760	0.336
Coverage	0.994±0.001	0.994±0.000	0.995±0.001	0.329	0.961	0.424

Table S10. Changes in the abundance of microbiota in the colon digest (%).

Team	Co_DA	Co_DB	Co_DC	P-value
Phylum				
Firmicutes	70.76±0.437	68.86±2.548	72.33±2.806	0.455
Bacteroidota	25.57±0.024	27.69±2.002	24.30±0.764	0.135
Spirochaetota	0.919±0.459	1.168±0.565	1.214±1.370	0.847
Verrucomicrobiota	1.200±1.325	0.747±0.572	0.993±0.766	0.850
Genus				
norank_f__norank_o__Bacteroidales	1.703±0.265[a]	1.067±0.529[ab]	0.318±0.151[b]	0.008
Candidatus_Saccharimonas	0.481±0.093[a]	0.100±0.037[b]	0.119±0.141[b]	0.018

Abbreviations: Co_DA = Bacteria in the cecum digestion of goats in group A; Co_DB = Bacteria in the cecum digestion of goats in group B; Co_DC = Bacteria in the cecum digestion of goats in group C.
At the phylum level, at least one group of bacteria listed has an abundance greater than 1%.
At the genus level, the bacteria has a significant change and at least one group has an abundance greater than 0.1%.
Values are expressed as Mean ± Standard Deviation.
"[a, b, c]" represent significant changes. Different letters represent significant changes between groups.

Table S11. Comparison of the copy number of functional bacterial species in colon under different nutrition levels

(lg(copies/g))

Team	Co_DA	Co_DB	Co_DC	*P* value
Butyrivibrio fibrisolvens	6.731±0.738	7.451±0.365	7.383±0.452	0.275
Fibrobacter succinogenes S85	5.255±0.561	7.315±0.353	4.853±1.130	0.015
Ruminococcus albus 7	7.023±0.179	7.481±0.056	7.939±0.380	0.038
Ruminococcus flavefaciens FD-1	7.023±0.103	7.480±0.031	7.090±0.108	0.011
Prevotella brevis GA33	6.630±0.034	6.548±0.052	6.425±0.143	0.080
Prevotella ruminicola 23	6.279±0.513	6.710±0.253	6.594±0.141	0.341
Ruminobacter amylophilus	5.320±0.531	5.610±0.098	5.497±0.180	0.582
Succinivibrio dextrinosolvens	6.877±0.054	6.893±0.189	7.195±0.523	0.446
Selenomonas ruminantium D	7.473±0.386	7.660±0.236	7.850±0.149	0.316

Table S12 Compare α diversity of the colon mucosa under different nutrition levels.

Team	Co_MA	Co_MB	Co_MC	*P* value(B-C)	*P* value(A-B)	*P* value(A-C)
Sobs	720.0±62.75	901.7±144.7	616.7±108.0	0.052	0.117	0.225
Shannon	3.942±0.147	4.139±0.806	3.339±0.686	0.261	0.700	0.210
Simpson	0.089±0.017	0.118±0.124	0.140±0.040	0.777	0.710	0.111
Ace	777.1±84.51	1090±154.7	678.8±130.3	0.024	0.037	0.334
Chao	774.5±72.92	1104±153.4	680.2±133.5	0.023	0.028	0.344
Coverage	0.997±0.001	0.993±0.000	0.997±0.002	0.023	0.005	0.970

microorganisms

Table S13. Changes in the abundance of microbiota in the colon mucosa (%)

Team	Co_MA	Co_MB	Co_MC	P-value
Phylum				
Firmicutes	33.17±2.849	43.73±11.19	21.34±13.93	0.288
Spirochaetota	35.57±6.632	38.53±16.71	21.67±23.44	0.665
Proteobacteria	10.89±4.166	0.340±0.258	29.09±12.20	0.037
Bacteroidota	13.79±2.392	14.87±7.221	6.912±3.781	0.162
Deferribacterota	0.087±0.046	0.006±0.010	12.90±13.39	0.128
Actinobacteriota	4.560±1.716	0.236±0.215	5.474±4.432	0.062
Campilobacterota	0.731±1.069	0.134±0.151	1.449±2.366	0.556
Genus				
norank_f__Mitochondria	9.538±4.637^{ab}	0.048±0.081^{b}	24.82±10.06^{a}	0.045
norank_f__Ruminococcaceae	0.578±0.284^{ab}	0.854±0.168^{a}	0.152±0.014^{b}	0.020
norank_f__norank_o__Izemoplasmatales	0.571±0.172^{a}	0.563±0.066^{a}	0.228±0.080^{b}	0.019
unclassified_f__Ruminococcaceae	0.139±0.057	0.347±0.046	0.196±0.213	0.032
Coprococcus	0.252±0.060^{a}	0.113±0.109^{ab}	0.050±0.038^{b}	0.034
Papillibacter	0.081±0.049^{ab}	0.155±0.037^{a}	0.027±0.028^{b}	0.033
norank_f__Christensenellaceae	0.032±0.016^{b}	0.109±0.012^{a}	0.011±0.020^{b}	0.004

Abbreviations: Co_MA = Bacteria in the cecum digestion of goats in group A; Co_MB = Bacteria in the cecum digestion of goats in group B; Co_MC = Bacteria in the cecum digestion of goats in group C.

At the phylum level, at least one group of bacteria listed has an abundance greater than 1%.

At the genus level, the bacteria has a significant change and at least one group has an abundance greater than 0.1%.

Values are expressed as Mean ± Standard Deviation.

“$^{a, b, c}$” represent significant changes. Different letters represent significant changes between groups.

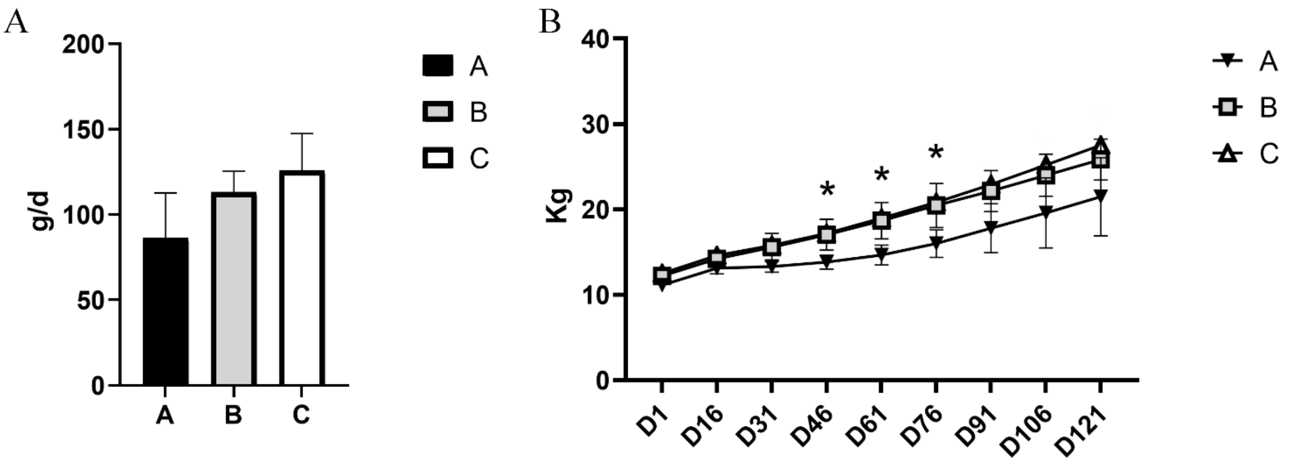

Figure S1. Effects of different feeding rates on growth performance. (**A**) Average daily gain (ADG), (**B**) Lamb weight at different times. "*" indicates 0.01< *p* <0.05.

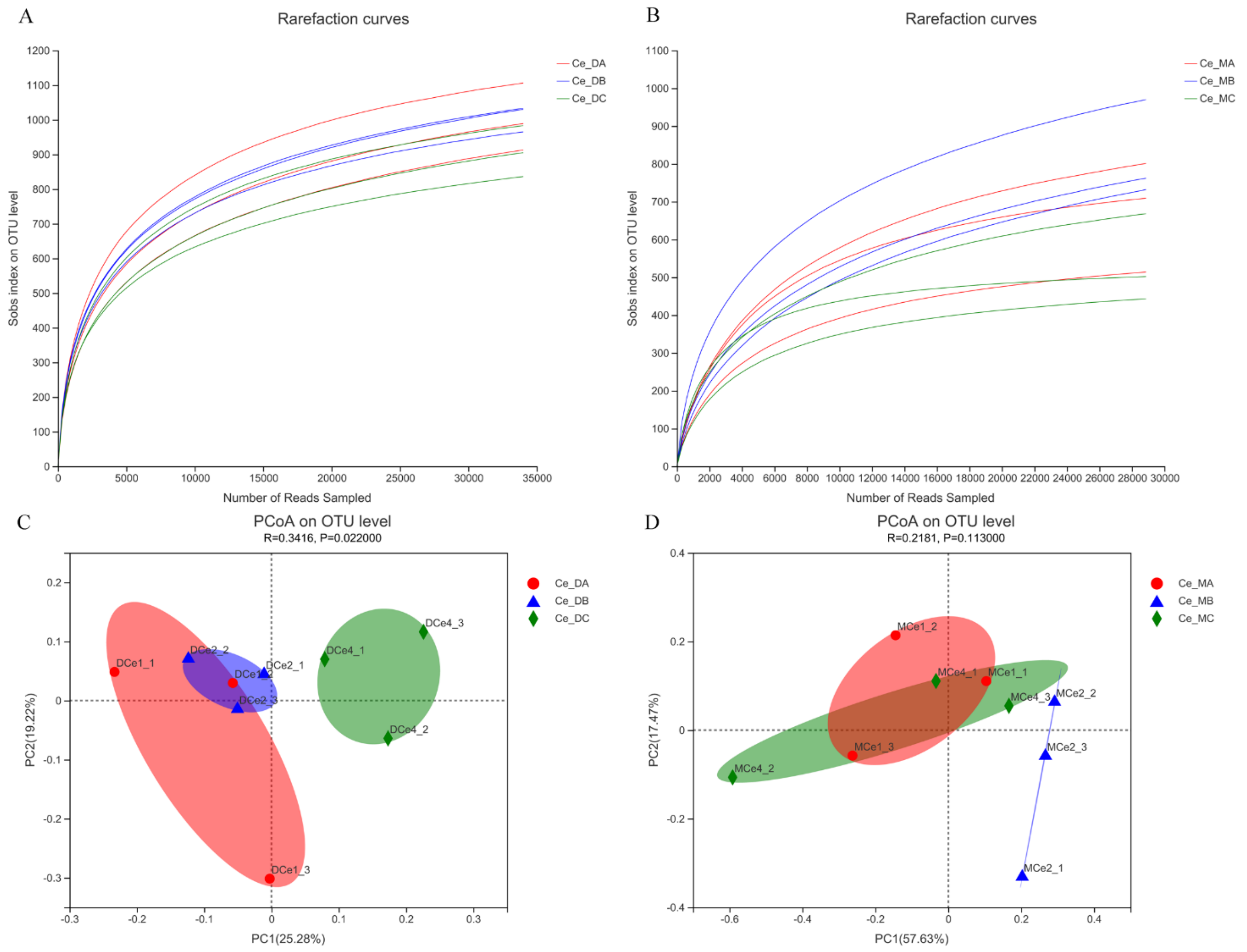

Figure S2. Refraction curves of bacteria in the (**A**) cecum digest and (**B**) cecum mucosa. PCoA of the microbial community in the (**C**) cecum digest and (**D**) cecum mucosa.

microorganisms MDPI

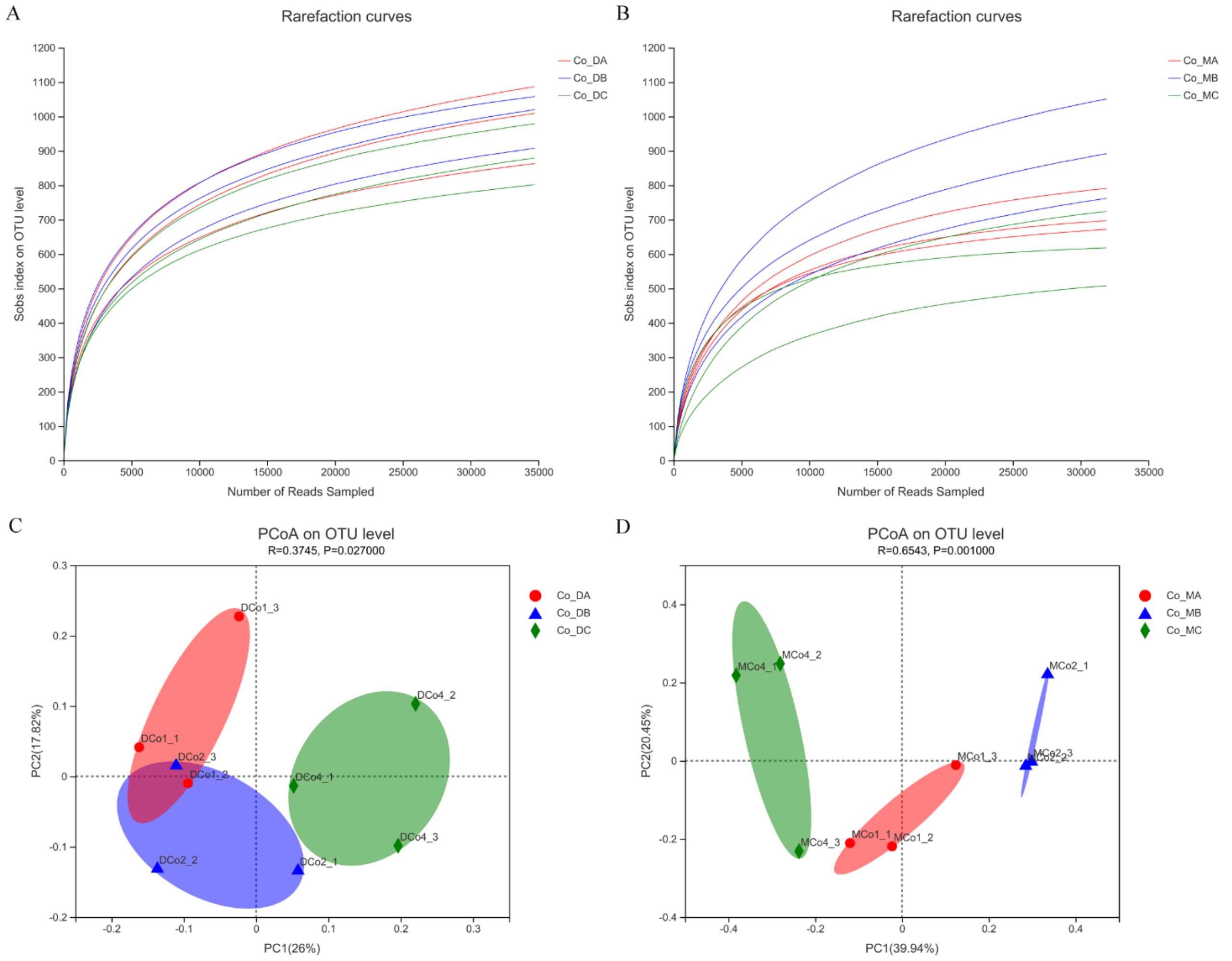

Figure S3. Refraction curves of bacteria in the (**A**) colon digest and (**B**) colon mucosa. PCoA of the microbial community in the (**C**) colon digest and (**D**) colon mucosa.
